# Reducing disease burden and health inequalities arising from chronic disease among indigenous children: an early childhood caries intervention in Aotearoa/New Zealand

**DOI:** 10.1186/1471-2458-13-1177

**Published:** 2013-12-13

**Authors:** John R Broughton, Joyce Te H Maipi, Marie Person, W Murray Thomson, Kate C Morgaine, Sarah-Jane Tiakiwai, Jonathan Kilgour, Kay Berryman, Herenia P Lawrence, Lisa M Jamieson

**Affiliations:** 1 Department of Preventive and Social Medicine, Dunedin School of Medicine, The University of Otago, 9054 Dunedin, New Zealand; 2 Raukura Hauora O Tainui Trust, The Base, Te Rapa, PO Box 101–30, 3241 Hamilton, New Zealand; 3 Raukura Hauora O Tainui Trust, South Auckland Mail Centre, PO Box 97644, Auckland, New Zealand; 4 Sir John Walsh Research Institute, Faculty of Dentistry, The University of Otago, PO Box 647, 9054 Dunedin, New Zealand; 5 Dental Epidemiology and Public Health Group, Faculty of Dentistry, The University of Otago, PO Box 647, 9054 Dunedin, New Zealand; 6 Waikato-Tainui College for Research and Development, 451 Old Taupiri Road, Hopuhopu, Private Bag 542, 3742 Ngaaruawaahia, New Zealand; 7 School of Dentistry, University of Toronto, Toronto, Canada; 8 Australian Research Centre for Population Oral Health, School of Dentistry, University of Adelaide, Adelaide, Australia

**Keywords:** Indigenous, Māori, Child, Mother, Oral health, Early childhood caries

## Abstract

**Background:**

Maaori are the Indigenous people of New Zealand and do not enjoy the same oral health status as the non-Indigenous majority. To overcome oral health disparities, the life course approach affords a valid foundation on which to develop a process that will contribute to the protection of the oral health of young infants. The key to this process is the support that could be provided to the parents or care givers of Maaori infants during the pregnancy of the mother and the early years of the child. This study seeks to determine whether implementing a kaupapa Maaori (Maaori philosophical viewpoint) in an early childhood caries (ECC) intervention reduces dental disease burden among Maaori children. The intervention consists of four approaches to prevent early childhood caries: dental care provided during pregnancy, fluoride varnish application to the teeth of children, motivational interviewing, and anticipatory guidance.

**Methods/design:**

The participants are Maaori women who are expecting a child and who reside within the Maaori tribal area of Waikato-Tainui.

This randomised-control trial will be undertaken utilising the principles of kaupapa Maaori research, which encompasses Maaori leadership, Maaori relationships, Maaori customary practices, etiquette and protocol. Participants will be monitored through clinical and self-reported information collected throughout the ECC intervention. Self-report information will be collected in a baseline questionnaire during pregnancy and when children are aged 24 and 36 months. Clinical oral health data will be collected during standardised examinations at ages 24 and 36 months by calibrated dental professionals. All participants receive the ECC intervention benefits, with the intervention delayed by 24 months for participants who are randomised to the control-delayed arm.

**Discussion:**

The development and evaluation of oral health interventions may produce evidence that supports the application of the principles of kaupapa Maaori research in the research processes. This study will assess an ECC intervention which could provide a meaningful approach for Maaori for the protection and maintenance of oral health for Maaori children and their family, thus reducing oral health disparities.

**Trial registration:**

Australia and New Zealand Clinical Trials Register (ANZCTR): ACTRN12611000111976.

## Background

Kaupapa Maaori is about recognising the strengths and aspirations of Maaori, along with Maaori rights to self-determination [1]. Bishop [2,3] advocated that kaupapa Maaori research must be founded on the principles of self-determination, legitimacy and authority, and empowerment for Maaori. Processes, procedures and consultation need to be correct so that, in the end, everyone who is connected with the research project is enriched, empowered, enlightened and glad to have been a part of it [4]. This study is being conducted according to the principles and practices of kaupapa Maaori research [5].

Oral health is an integral component of overall health and wellbeing. Dental caries among children is a chronic condition that not only causes pain, but impacts on a child’s ability to function (Sheiham [6], Gaynor and Thomson [7], Foster Page et al. [8]). ECC is defined as the presence of one or more decayed, missing or filled teeth (dmft, the index for caries severity) in the primary dentition in a child younger than six years. In children younger than 3 years, any sign of dental decay is considered to be severe ECC and may impact on their future oral health. ECC is preventable, in theory.

Across all age groups Maaori do not enjoy the same oral health status as non-Maaori. The Dunedin Multidisciplinary Health and Development Study is a cohort study of 1,000 babies born in Dunedin in 1972. In work with that study, Koopu [9] concluded that ‘for a cohort of New Zealanders followed over their life course, the oral health features of caries prevalence, caries severity and periodontal disease prevalence are higher among Maaori than non-Maaori.’ Thomson [10] reported that 5-year-old Maaori children in the Manawatuu-Wanganui Area Health Board were three times more likely than non-Maaori children to have high experience of dental caries; Maaori children were three times more likely to have undergone general anaesthesia for dental treatment; and Maaori children were over three times more likely not to have been enrolled in the School Dental Service (SDS) prior to starting school. The report to the Minister of Health on child oral health inequalities in New Zealand (Thomson et al. [11]) stated that, “There is increasing concern that an epidemic of dental decay in certain parts of New Zealand is impacting negatively on certain groups of New Zealanders, namely Maaori and Pacific people, and people from low socioeconomic status groups.”

### ECC intervention rationale

The literature suggests four ways in which ECC can be successfully prevented:

(1) Dental care provided to the mother during pregnancy (Li et al. [12]). Provision of comprehensive dental care to mothers during pregnancy reduces their levels of Streptococcus mutans, a micro-organism associated with ECC that can be transferred to the infant at birth.

(2) Fluoride varnish application to children’s teeth. The application of topical fluoride varnish has been shown to be effective in the prevention of ECC, with Weintraub et al. [13] reporting little difficulty with compliance and no adverse events.

(3) Anticipatory guidance (Nowak and Casamassimo [14]) is a pro-active, developmentally-based counselling technique that focuses on the needs of a child at a particular stage of life.

(4) Motivational interviewing (Harrison et al. [15]) on the other hand, focuses on strategies to move carers from inaction to action, with many possible paths to a solution provided.

### Aim and hypotheses

This study seeks to determine whether implementing a kaupapa Maaori ECC intervention reduces the dental disease burden and oral health inequalities among Maaori children living in the Waikato-Tainui tribal area of Aotearoa/New Zealand. We hypothesize that the implementation of a kaupapa Maaori ECC intervention will reduce the dental disease burden among Maaori children. This research project is being conducted in partnership with Indigenous oral health research groups in Australia and Canada.

## Methods/design

### Intervention review

**Figure 1:**
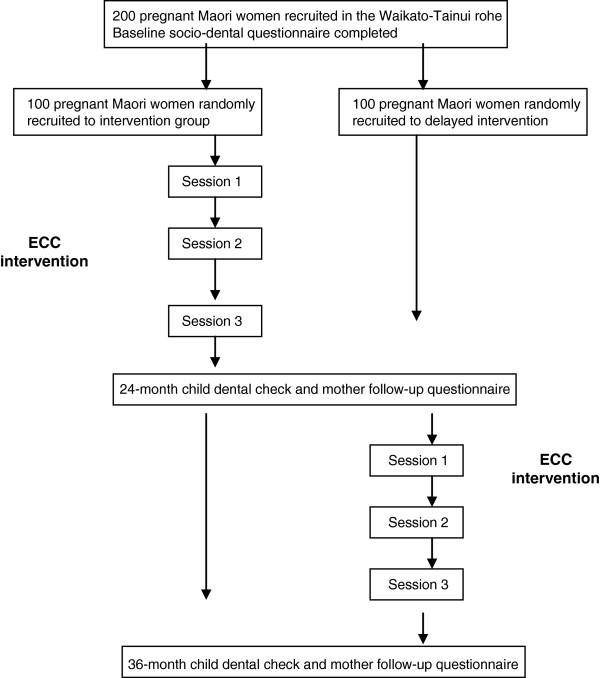
Study design schema.

This is a randomised, controlled trial in which participants are offered four intervention components: (1) provision of dental care during pregnancy; (2) fluoride varnish application to the teeth of children; (3) motivational interviewing and (4) anticipatory guidance. A schema outlining the study design is presented in Figure 1.

1. Dental care

In the intervention group, dental care is arranged during pregnancy or when the participant is able to attend for dental care. For participants in the delayed intervention group, dental care is also arranged at this same time. The basic dental care provided includes examination, radiographs, relief of pain, control of infection, scaling and prophylaxis, elimination of caries, restorations, and extractions. This level of care has been accounted for in the research budget, and additional funding can be arranged through Work and Income New Zealand (WINZ) if the participant is eligible for this benefit. The Maaori research assistants working on this project liaise with the participants and their whaanau (family) to ensure that the participants are able to attend the dental clinic for their dental care.

2. Fluoride varnish applications

In New Zealand, the application of fluoride varnish is a clinical procedure that can be applied only by a registered oral health professional (either a dentist, a school dental therapist or a dental hygienist). In the intervention group, varnish application occurs when children are aged 6-, 12- and 18-months. Children in the delayed intervention group receive fluoride varnish applications at 24-, 30- and 36 months.

3. Motivational interviewing

Motivational interviewing (MI) within a Kaupapa Maaori context is a culturally adaptive model in that it honours and respects whakaute, the beliefs and core values of individuals, groups, organisations and communities (Miller and Rollnick [16]). MI aligns well with Indigenous cultures in that it is consistent with the life-ways and values of people within these cultures. Miller and Rollnick [16] stated that “we listen more than we tell, we take away power differentials/hierarchy and work collaboratively.” The foundation of MI is to listen fully to an individual (or community, etc.) in order to gain a sincere understanding of the person sitting with us (we gain an understanding of their context, what values they hold, their thoughts, feelings, and behaviour, and what is important to that person) i.e. hinengaro or the mental/emotional dimension of a person’s existence.

MI is used as a way to understand where people are coming from, their whakapapa (genealogy) and to talk about potential changes (behaviour changes, organisational changes, practice changes). The MI practitioner does not suggest change, does not force conversation, and does not judge people based on their desire, or lack of, to change. If the person desires change, then the MI practitioner and the individual (community, etc.) work together (which is referred to in Maaori as mahi tahi) to define what the change will look like. The key component is that, for Maaori, MI embodies whanaungatanga or relationships in that we are all one and we are walking the journey together.

Each participant will receive oral-health-related items consistent with three specific themes. These will comprise the relevant Anticipatory Guidance (AG), Maaori-focused oral health promotion material on basic oral care behaviours, the New Zealand oral health care system, fluoridation and oral health aids (toothbrushes, fluoride toothpaste).

The three themes covered by each MI/AG phase are: firstly, oral health knowledge, which encompasses teeth eruption and teething, reasons for childhood dental diseases, ways childhood dental disease can be prevented, foods and beverages harmful for oral tissues, behaviours harmful for the oral tissues, and the oral health and general health relationship; secondly, oral self-care, which encompasses ways to look after children‘s teeth, use of toothbrush, toothpaste and disclosing solution, and use of oral health services; and thirdly, oral health protection and community water fluoridation. This will incorporate oral health as a part of “Te Whare Tapa Whā”, which is a model of Maaori health and well-being. This model recognises the four dimensions of tinana (the physical dimension), hinengaro (the mental and emotional dimension), whaanau (the family or social dimension), and wairua (the spiritual dimension).

Participants in the delayed intervention group receive the motivational interviews when the child is aged 24-, 30-, and 36 months.

4. Anticipatory guidance

Anticipatory guidance is carried out in alignment with the motivational interviewing sessions.

#### *Participants and recruitment*

Participants are expectant Maaori mothers residing within the Waikato-Tainui tribal area. This is because the Maaori health provider, Raukura Hauora O Tainui, has extensive primary health care services throughout its tribal area, and close networking relationships with other health providers and services. The interventions are timed differently (the intervention group begins in pregnancy, while the delayed intervention group begins their interventions when the child is aged 24- months). However, all participants are offered free basic dental care on recruitment. Support is also provided for participants to access the dental clinic and oral health packs including toothbrushes and toothpaste are provided for them. Recruitment commenced on 1 July 2011 and will end on 31 December 2012.

#### *Ethics and consent*

This study received approval from the Northern Y Ethics Committee, which is under the auspices of the Waikato District Health Board. The Ethics Application was supported by the Waikato-Tainui kaumātua (elders) group of the Waikato District Health Board.

#### *Maaori cultural framework*

This study is being conducted within the North Island Tribal area of Waikato-Tainui and is the responsibility of two tribally derived organisations: Raukura Hauora O Tainui and the Waikato-Tainui College for Research and Development. Both organisations are working in partnership with a research team from the Faculty of Dentistry of the University of Otago. All of the Waikato-Tainui based researchers are Maaori and, as such, adhere to the principles and procedures that are governed by their respective Boards. According to Smith [17], many indigenous communities still possess ancient or traditional memories and other ways of knowing that informs many of their contemporary practices. In order to be relevant to Waikato-Tainui, “Te Niho Taniwha” has been identified as a tribal model that provides a way for kaupapa Maaori to be viewed within a Waikato-Tainui context. Te Niho Taniwha is a cultural framework that is relevant to Waikato-Tainui, as a way of organising values, thoughts and actions that guide and support the customs and way of life of Waikato Tainui.

**Figure 2:**
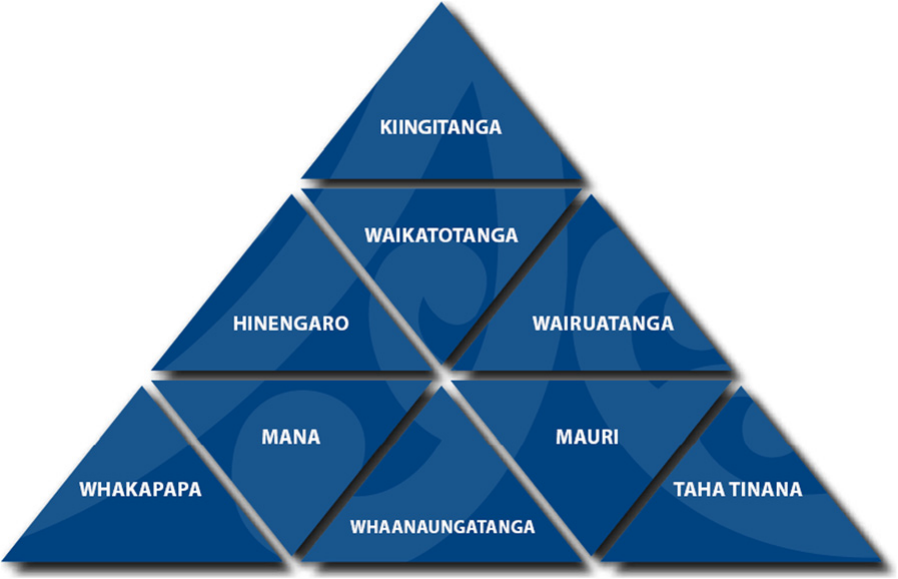
Model of Te Niho Taniwha.

It is important to describe in some detail the meaning of “Te Niho Taniwha” framework and particularly the application of its meaning to the study. “Te Niho Taniwha” framework is, for Waikato-Tainui, central to the kaupapa Māori methodology being applied to the study; it gives meaning and significance to the work being undertaken (Figure 2). Te Niho Taniwha draws on a set of values, principles, philosophy and practice that is iwi-derived and grounded firmly in Waikato-Tainui maatauranga (knowledge). With its strong foundation and three equal sides, the symbol of the Niho Taniwha shows strength and resilience; it also illustrates the elements needed to attain total wellbeing.

Considered alone, each niho (tooth)—which is represented by an individual triangle in the model—is one fundamental element of the cultural framework but, together, they represent wellbeing as a people, as an iwi (tribe), as hapuu (sub-tribe) and as a whaanau (family). Together, they mean much more than the sum of the individual parts. More broadly, the niho taniwha also culturally locates and positions the framework and the study itself within the heartland of Waikato-Tainui, drawing on cultural symbolism of the taniwha as an ancestor of the major water body of the tribe, the Waikato River.

### Kiingitanga

The Kiingitanga is the overarching niho of Te Niho Taniwha and is especially significant to Waikato-Tainui people. It alludes to the pan-tribal movement that was established in 1858 which sought to unite tribes around New Zealand and which Waikato-Tainui retain guardianship of.

### Waikatotanga

Underneath the Kiingitanga is Waikatotanga. This refers specifically to the Waikato-Tainui tribe and is located there primarily to remind us of the past and to ensure that we keep to the cultural practices of Waikato-Tainui. Its location underneath the Kiingitanga is also a reference to the role of the tribe as guardians of the Kiingitanga, which reinforces the importance of keeping hold of tribal cultural practices.

### Hinengaro

Hinengaro refers to our thoughts, feelings and behaviours. It is the seat of our emotions and is often sensed deep down in the puku (stomach). It can be referred to the ability for a person to think through situations.

### Wairuatanga

Wairuatanga (or spirituality) is exemplified in the whakatauiiki: *Kei roto i te kapu o tooku ringa enei taonga, homai tou ka whaa, kapi katoa.* (The spirit of the people comes from the heavens). No matter who they are, people are sacred. From infants, children, and adolescents to elders; all are sacred.

### Whakapapa

Whakapapa (genealogy) defines who we are as people. It links us to our ancestors, defines our heritage and defines our place in the world. Mana Tupuna (Ancestral authority) helps us know who we are, from whom we descend, and what our obligations are to those who come after us. Whakapapa is also a tool utilised in analysing and synthesising information and knowledge.

### Mana

Mana refers to an extraordinary power, essence or presence. This applies to the energies and presences of the natural world. There are degrees of mana and our experiences of it, and life seems to reach its fullness when mana comes into the world. The most important mana comes from Te Kore, the realm beyond the world we can see, and sometimes thought to be the ‘ultimate reality’.

### Whanaungatanga

Whanaungatanga (kinship ties) underpins the social organisation of whaanau (family), hapuu (sub tribe) and iwi (tribe) and includes rights and reciprocal obligations consistent with being part of a collective. It is the principle which binds individuals to the wider group and affirms the value of the collective. Whanaungatanga is inter-dependence with each other and recognition that the people are our wealth.

### Mauri

Mauri is an energy which binds and animates all things in the physical world. Without mauri, mana cannot flow into a person or object. Mauri is crucial to the well-being of relationships and issues (kaupapa). It informs how and why activities should be undertaken and monitors how well these are progressing towards their intended goals.

### Taha Tinana

Taha tinana in its most basic sense is the physical body. It incorporates the physical form and its emotional components.

These entire elements combine together to make up the *Niho Taniwha*. In isolation, each element has its own function and purpose, but collectively, come together to provide a common sense of purpose and provides a call to action. Durie [18] notes that such an approach draws on maatauranga Maaori (as an underlying traditional body of knowledge) to inform and provide an approach to the research that is distinctly Maaori. Kaupapa Maaori research describes this type of approach as agenda setting which has as its focus indigenous wellbeing, development and advancement as core foci. Smith [19] argues that kaupapa Maaori must be more than a theoretical exercise and find ways to address “in practical terms our own issues in our own communities” (p. 14). These fundamental considerations have informed the way in which the project has been approached what Linda Smith has labelled the ‘tricky ground’ of research.

Potential participants are invited into the study through the extensive tribal health and social services of Raukura Hauora O Tainui. The kaimahi (research assistants) discuss the project with potential participants kanohi-ki-te kanohi (face-to-face) and provide them with the panui (information sheet) about the project. If the are agreeable to participate the consent form is explained and duly signed. The participants are informed that they may withdraw at any time they so wish and they do not have to provide an explanation. They are provided with the contact details of the researchers so that they may contact them at any time about any aspects of the project.

Five Maaori staff have been employed for the study and are located within either of the two Waikato-Tainui organisations, Raukura Hauora O Tainui or the Waikato-Tainui College for Research and Development.

The Aotearoa/New Zealand arm of this international collaborative grant has been funded by Te Kaunihera Rangahau Hauora O Aotearoa The New Zealand Health Research Council (HRC ICIHRP Grant application ID 09/644).

### Data collection

Clinical and self-reported data collected during interventions are entered into Microsoft Excel and Access databases, and stored securely at the Waikato-Tainui College for Research and Development.

#### *Sample size*

A sample size of 192 participants (96 per study arm; alpha = 0.05, power = 90 percent, 50 percent attrition) was calculated as being necessary to detect ECC incidence differences, based on reports of ECC incidence among Indigenous children in the literature (50–75 percent over two years). For convenience, this was rounded up to 200 participants; 100 in the intervention group and 100 in the delayed intervention group.

#### *Allocation to groups*

Upon agreeing to take part, each mother is randomly allocated to either the Intervention Group or the Delayed Intervention Group by choosing an envelope which contains the name of the group. The initial plan to use block randomisation was not implemented owing to a procedural error.

#### *Questionnaires*

On recruitment, a baseline questionnaire is completed which includes self-reported information on their socio-demographic status, general health status, dental health status, dental behaviours and perceptions. The short-form Oral Health Impact Profile (OHIP-14; Slade [20]) is used to collect information on respondents’ oral-health-related quality of life (OHRQoL). This comprises 14 items, two for each of seven domains (functional limitation, physical pain, psychological discomfort, physical disability, psychological disability, social disability and handicap). Responses are coded as ‘Very often’ (scoring 4), ‘Fairly often’ (3), ‘Occasionally’ (2), ‘Hardly ever’ (1) or ‘Never’ (0). OHIP-14 scores were computed in two ways: first, an overall OHIP-14 score was calculated by summing responses over all 14 items. The total OHIP-14 score is a measure of the ‘severity’ of adverse impacts caused by oral conditions, and uses all response categories. Second, the prevalence of impacts are computed (at subscale and whole-scale level) by identifying individuals who experienced impacts ‘Very often’ or ‘Fairly often’. Use of the OHIP-14 will be repeated when the children are aged 24 and 36 months.

Covariates include socio-demographic characteristics, and self-reported oral health and general health. Socio-demographic characteristics will include maternal age, *iwi* (tribal) affiliation, residential location, occupation, educational status and state financial support. Oral health covariates will include self-reported oral health, oral health-related quality of life, and history and experience of dental services of both the mother and child. General health covariates will include mother behaviours such as tobacco smoking, alcohol consumption, current medication, current diagnoses (for diabetes etc.), status of current diagnoses (controlled, uncontrolled etc.) and self-reported general health.

#### *Clinical examinations*

After enrolment in this project, some basic dental treatment will be provided for the mother which will include examination and radiographs, scaling and cleaning, non-complicated extractions, and basic dental restorations.

Information about child clinical oral health status will be collected during standardised examinations conducted at the 24- and 36-months follow-ups by two calibrated dental professionals. Before examination, a new toothbrush will be used to clean the teeth, and the teeth dried with gauze. Examiners will follow a standardised protocol. Procedures appropriate for young children will be used during the examinations; for example, children will be examined in the ‘knee-to-knee’ position on their mother’s lap. Standard cross-infection control procedures will be followed, and a fibre-optic light used as a light source. No sharp instruments will be used during examinations. The status of all teeth and tooth surfaces will be examined and recorded. Non-cavitated and cavitated lesions will be recorded for all teeth. The study will focus on the earliest manifestation of ECC. Two ECC case definitions will be used: (1) one or more upper incisor teeth labial surfaces being carious, either non-cavitated or cavitated, and; (2) one or more non-cavitated or cavitated carious, missing or filled surfaces. Lesions will be diagnosed as ‘non-cavitated’ if an area of demineralization is without loss of surface continuity detected visually. Lesions will be diagnosed as ‘cavitated’ if a loss of continuity of the enamel is detected (Drury et al. [21]). In addition, levels of tooth loss and dmft/s indices will be assessed from the tooth- and surface-level recorded observations. Diagnosis will be based on visual criteria only.

### Statistical analysis

The data will be analysed according to intention-to-treat. A mixed model, using a random effect for participant, will be used to analyse the data. This will compare the ECC-related outcomes between the early intervention group and the delayed intervention group at both 24 and 36 months. This model takes into account the correlation between the repeated measures and copes reasonably well with missing values provided they are missing at random. Some sensitivity analysis will be carried out to examine the effect of missing data. The results will be presented as differences and 95% CI. Two aspects will be focused upon in the analyses: (a) within-individual changes and (b) between-individual changes in the primary outcomes of child ECC experience and carer oral health knowledge, oral self-care, dental service utilisation, oral health-related self-efficacy and oral health literacy. The Generalised Linear Mixed Model (GLMM) approach will be used (using STATA statistical software) to take account of the study’s cross-over design. Aside from the baseline comparison, there will be two main analyses. First, after 24 months, there will be a comparison between the intervention and delayed intervention groups, assessing the effect of the ECC intervention on ECC-related outcomes. Second, there will be a comparison between the 24- and 36-month intervention group ECC-related outcomes, assessing the effect of the intervention after 36 months, and including terms for both group and period. All participants with at least one set of follow-up ECC-related outcome data will be included in multivariate modelling. Analysis will include fitting GLMM models for analysis of the correlated data, in order to determine factors associated with ECC-related changes.

## Discussion

Early childhood caries remains a major health issue in New Zealand, even though dental treatment for all New Zealanders is free until their 18th birthday. The reasons for ECC disparities are multi-factorial including diet, oral health behaviours, exposure to fluoridation, social determinants of health, health service delivery and access to oral health services. ECC can be successfully prevented by fluoride varnish application to the teeth of children, and the engagement of the mother through the provision of dental care during pregnancy together with anticipatory guidance and motivational interviewing. Provision of dental care to mothers during pregnancy reduces their levels of *Streptococcus mutans*, a micro-organism associated with ECC that can be transferred to the infant at birth, while topical fluoride varnish has been shown to be efficacious in ECC prevention with little difficulty with compliance and no adverse events. Anticipatory guidance is a developmentally-based counselling technique that focuses on the needs of a child at a particular life stage, while motivational interviewing focuses on strategies to move carers from inaction to action, with many possible paths to a solution provided. To date there has been no single initiative reported that has adopted all four of these intervention strategies which is what our study proposes to do.

This research is being undertaken as a collaboration between Indigenous health researchers in New Zealand, Australia and Canada with, for and by the respective Indigenous populations. The World Health Organization view is that ‘health research involving Indigenous Peoples, whether initiated by the community itself or by a research institute, needs to be organised, designed and carried out in a manner that takes account of cultural differences, is based on mutual respect, and is beneficial and acceptable to both parties’ (Sims et al. [22]). As such, this study is being carried out according to the practices and principles of kaupapa Maaori research. It is only by engaging in this approach that the outcomes will have validity, credibility and acceptability to Maaori and will have the potential to make a significant contribution to the improvement of Maaori health status and overall Maaori health.

## Abbreviations

AG: Anticipatory guidance; dmft/s: Decayed, missing or filled teeth/surfaces in the primary dentition; ECC: Early childhood caries; GLMM: Generalized Linear Mixed Model; MI: Motivational interviewing; MS: Mutans streptococci.

## Competing interests

The authors declare that they have no competing interests.

## Authors’ contributions

JB, JM, MP, WMT, and KM participated in the study design, and ethics application. S-JT, JM, JK, KB, and MP provided significant intellectual input regarding the kaupapa Maaori methodology and the principles and practices of Waikato-Tainui culture. WMT was responsible for the statistical methodology in the study design. KM was responsible for the oral health promotion aspects of the study design; MP coordinated data collection and data management. HL (Canada) and LJ (Australia) participated in the international Indigenous aspects of the study. All authors were involved in revising the manuscript for important intellectual content and read and approved the final manuscript.

## Pre-publication history

The pre-publication history for this paper can be accessed here:

http://www.biomedcentral.com/1471-2458/13/1177/prepub
